# Concentrations of Seven Phthalate Monoesters in Infants and Toddlers Quantified in Urine Extracted from Diapers

**DOI:** 10.3390/ijerph18136806

**Published:** 2021-06-24

**Authors:** Fiorella Lucarini, Marc Blanchard, Tropoja Krasniqi, Nicolas Duda, Gaëlle Bailat Rosset, Alessandro Ceschi, Nicolas Roth, Nancy B. Hopf, Marie-Christine Broillet, Davide Staedler

**Affiliations:** 1 Department of Biomedical Sciences, University of Lausanne, 1011 Lausanne, Switzerland; fiorella.lucarini@unil.ch (F.L.); marc.blanchard@unil.ch (M.B.); tropoja.krasniqi@unil.ch (T.K.); nicolas.duda@unil.ch (N.D.); marie-christine.broillet@unil.ch (M.-C.B.); 2 Scitec Research SA, Av. De Provence 18, 1007 Lausanne, Switzerland; gbailat@scitec-research.com; 3 Division of Clinical Pharmacology and Toxicology, Institute of Pharmacological Sciences of Southern Switzerland, Ente Ospedaliero Cantonale, 6900 Lugano, Switzerland; Alessandro.Ceschi@eoc.ch; 4 Faculty of Biomedical Sciences, Università della Svizzera Italiana, 6900 Lugano, Switzerland; 5 Department of Clinical Pharmacology and Toxicology, University Hospital Zurich, 8091 Zurich, Switzerland; 6 Swiss Centre for Applied Human Toxicology (SCAHT), University of Basel, 4055 Basel, Switzerland; nicolas.roth@unibas.ch (N.R.); nancy.hopf@unisante.ch (N.B.H.); 7 Center for Primary Care and Public Health (Unisanté), University of Lausanne, 1007 Lausanne, Switzerland

**Keywords:** phthalates, urine, endocrine disruptors, biomonitoring, children’s exposure, children’s health, emerging contaminants

## Abstract

Carrying out exposure studies on children who are not toilet trained is challenging because of the difficulty of urine sampling. In this study, we optimized a protocol for urine collection from disposable diapers for the analysis of phthalate metabolites. The exposure of Swiss children (*n* = 113) between 6 months and 3 years of life to seven phthalates was assessed by gas chromatography–mass spectrometry measurements. The study showed limited exposures to phthalates, with only 22% of the samples containing some of the metabolites investigated. The three most frequently detected metabolites were monoethyl phthalate, mono-cyclohexyl phthalate, and mono-benzyl phthalate. We also detected mono-*n*-octyl phthalate and mono(3,5,5-trimethylhexyl) phthalate, which have rarely been observed in urine from infants and toddlers; therefore, di-n-octyl phthalate and bis(3,5,5-trimethylhexyl) phthalate can be considered as potentially new emerging phthalates. This study presents an initial snapshot of the Swiss children’s exposure to phthalates and provides a promising approach for further phthalate biomonitoring studies on young children using disposable diapers as urine sampling technique.

## 1. Introduction

Phthalates are organic compounds used in a large variety of industrial applications as solvents, plasticizers, and additives [1]. Due to their widespread utilization, phthalates are present in many types of products such as food packaging, cosmetics, medical devices, toys, dentures, paints, adhesives, and nail polishes [1]. This class of compounds gives flexibility and durability to plastic products [2]. Phthalates are not chemically bound to the polymers and can leach out during use. Consequently, handling these phthalate-containing products can lead to significant exposures to phthalates [2]. 

Phthalate metabolites can act as agonist ligands or antagonists to hormone receptors, thus interfering with the endocrine-hormone-mediated pathways. Phthalate exposures have been linked to numerous reproductive, developmental, metabolic, respiratory, and attention deficit hyperactivity disorders (ADHD), in particular, decreased male fertility and preterm birth, and diabetes and insulin resistance, for which associations are strong [3,4,5,6,7,8,9,10].

Humans are exposed to phthalates by ingestion of contaminated beverages or foods, inhalation of air or dust and by skin contact [11]. Young children typically below 3 years old are more likely to be exposed to phthalates because they suck or chew plastic toys and other objects containing plasticized materials; in addition, they spend more time on the floor, which may increase their exposure to phthalate-contaminated dust originating from the building material [12].

The European Union regulated the use of seven phthalates in children’s toys and products (i.e., benzyl butyl phthalate (BBP), dibutyl phthalate (DBP), diisobutyl phthalate (DIBP), di-2-ethylhexyl phthalate (DEHP), diisodecyl phthalate (DIDP), diisononyl phthalate (DINP), and di-n-octyl phthalate (DNOP)), whilst Switzerland regulated the use of four phthalates (i.e., DEHP, DIBP, DBP, and BBP). Similar restrictions have also been adopted by the United States and Canada [13,14,15,16,17,18,19,20,21]. Despite these regulations, a large number of recent biomonitoring studies show the presence of these as well as the non-regulated phthalates (e.g., diethyl phthalate (DEP), dimethyl phthalate (DMP), and dicyclohexyl phthalate (DCHP)) in urine samples from the general population and, in particular, from children [22,23]. These studies quantified known phthalate metabolites in urine, which give a snapshot of current exposures because phthalate elimination half-lives are fairly short (less than 24 h) [24,25]. Phthalate exposures are detected in urine as monoesters or oxidized metabolites [26] in their conjugated form. These metabolites are suitable biomarkers to investigate current exposures to the parent compounds [27]. Phthalate metabolism efficiency differs strongly between children and adults, and the toxicokinetics depend on several factors such as the size of lipid and tissue compartments, organ blood flow, and protein binding capacity. Metabolism is also strongly affected by the immature function of renal and hepatic systems [28]. Therefore, young children will likely be much more affected than adults following longer exposure durations, which may lead to more harmful effects due to the still undeveloped detoxification functions [29]. Although this is known, the urinary concentrations of the phthalate metabolites in young children under 3 years of life remain poorly investigated. Only a few studies have investigated phthalate exposures during the early stages of life (from 0 to 36 months) [29,30,31,32,33,34]. These studies mainly quantified metabolites of DEP, DBP, DIBP, di-n-pentyl phthalate (DNPP), di-isopentyl phthalate (DIPP), BBP, DEHP, DNOP, DINP, and DIDP and among these, the highest concentration ranges (>1000 μg/L) were for the metabolites of DIBP, DBP, and BBP.

Children’s exposures to phthalates have been reported by a European biomonitoring study (2010–2012) including 17 countries called DEMOCOPHES (Demonstration of a Study to Coordinate and Perform Human Biomonitoring on a European Scale) [35]. The study investigated the presence of the metabolites of DEHP, DEP, BBP, DBP, and DIBP in urine samples obtained from children between 5 and 11 years old. Concerning the Swiss population investigated in this study, metabolites of DEHP were detected at a geometric mean (GM) concentration of 28.1 μg/L while other phthalate metabolites were found at GM levels between 5.1 and 20.5 μg/L. Younger children (5–8 years old) had higher levels compared to older ones (9–11 years old). Generally, urinary phthalate metabolites were found to be higher in children compared to that in adults. These results from the DEMOCOPHES study are similar to what has been reported by the U.S. NHANES (National Health and Nutrition Examination Survey) cohort study [36], where higher concentrations of DBP, BBP, and DEHP metabolites were observed in 6–11-year-old children compared to adults.

To the best of our knowledge, there is no study evaluating phthalate concentrations in children under 3 years of life in Switzerland. The main reason for this data gap can be linked to the infants’ and toddlers’ immaturity of bladder control, which makes urine sampling challenging. Diapers could, therefore, be a valid alternative collection medium among children in this age group [37]. There are not many biomonitoring studies to date that use diapers as a collection medium for the urine of non-toilet-trained children. In 2004, Hu et al. developed a urine extraction method from diapers to investigate the presence of pyrethroid pesticide metabolites [38]. Recently, interest in this sampling technique has grown, and a few studies have been published in this regard. In particular, urine extracted from diapers has been used to investigate the exposure of children to organophosphate insecticides [39], neonicotinoid insecticides [40], pyrethroid insecticides [41], tebuconazole [42], and phenolic endocrine disruptors [43] such as bisphenol A, bisphenol A analogs [44], triclosan, and phthalates [37]. These studies outlined several advantages on the use of diapers to collect urine in order to perform large surveys of young populations. For instance, it would be possible to sample a large number of urine samples without involving specialized workers for the sampling or without requiring additional help from the parents of the children. Additionally, the collection of urine over the entire day would be possible with diapers. 

Monoester phthalates are generally used as biomarkers in children as they have a short half-life in the body and are excreted quickly in urine. These monoesters are primary metabolites of the phthalate esters (Phase I biotransformation). In addition, several secondary and oxidative metabolites have been reported for DEHP and other long-chain phthalates. For instance, the main metabolites for DEHP are oxidative metabolites such as mono-(2-ethyl-5-oxohexyl)phthalate (MEOHP), mono-(2-ethyl-5-hydroxyhexyl)phthalate (MEHHP), mono-(2-ethyl-5-carboxypentyl)phthalate (MECPP), and mono-(2-carboxymethyl-hexyl)phthalate (MCMHP) [45]. The oxidative metabolites of other phthalates are, however, not well characterized.

The aim of this study was to assess exposures to seven phthalates (Table 1) in a small cohort of Swiss children between 6 months and 3 years of life, using disposable diapers as the urine collection method, and to quantify the urinary concentrations of their monoester metabolites. These seven phthalates were selected to get an overview of the exposures to phthalate belonging to three groups: (i) regulated (i.e., BBP, DBP, DEHP, DNOP), (ii) used without restriction (i.e., DCHP, DEP), and (iii) poorly studied (bis(3,5,5-trimethylhexyl) phthalate (DTMHP)). To assess diapers as biomonitoring sampling methods, for simplicity, the study focused only on monoester metabolites while also attempting to include a large variety of phthalates.

## 2. Materials and Methods

### 2.1. Study Population

Disposable diapers containing the urine were collected in daycare centers from three cantons in Switzerland widely distributed in the three main language area. In particular, the samples were collected from four different daycare centers located both in metropolitan and rural areas.

The age of the investigated population is that of children attending daycare centers, i.e., between 6 months and 3 years (i.e., infants (6 months to 1 year), toddlers (1 to 2 years), and young children (2 to 3 years) [46]). Parental consent was obtained for 113 children (67% boys and 33% girls with a mean age of 20.2 months). Gender and age for each child were the only parameters known to the investigators.

### 2.2. Sample Collection

Our biomonitoring study was based on a single spot urine sample per child extracted from diapers collected between 9 a.m. and noon. This study was conducted between June and July (2019), and samples were stored at −20 °C immediately after sampling until analysis. Sample collection did not require approval by the competent ethics committee (i.e., the canton of Vaud’s ethical committee (CER-VD)) because the samples were anonymized and were considered waste material by CER-VD.

### 2.3. Chemicals

Stock standard solutions containing the seven metabolites investigated (i.e., monoethyl phthalate (MEP); mono-benzyl phthalate (MBzP); mono-butyl phthalate (MBP); mono-cyclohexyl phthalate (MCHP); mono-2-ethylhexyl phthalate (MEHP); mono(3,5,5-trimethylhexyl) phthalate (MTMHP); and mono-n-octyl phthalate (MnOP) (100 mg/L in cyclohexane)) were supplied by Neochema GmbH (Germany). Working solutions for calibrations were prepared by serial dilution of the stock standard mixture with acetonitrile (Sigma-Aldrich, Darmstadt, Germany) Beta-glucuronidase from *Escherichia coli* (200 units/mL) was purchased from La Roche AG group. BSTFA (N,O-bis(trimethylsilyl)trifluoroacetamide) with 1% of TMCS (trimethylchlorosilane) was purchased from Sigma-Aldrich. Ammonium acetate (98%), calcium chloride (CaCl_2_) (≥99%), creatinine hydrochloride (≥97%), ammonium phosphate dibasic (≥99%), magnesium chloride hexahydrate (≥99%), potassium chloride (≥99%), sodium sulfate (≥99%), urea (≥98%), acetone (99.9%), formic acid (≥95%), and methanol (99.9%) were purchased from Sigma-Aldrich. Nanopure water was provided by an ultrapure water system (Arium Pro, Sartorius, Göttingen, Germany).

### 2.4. Sample Preparation and Extraction

Although diapers represent a promising sampling method for biomonitoring studies, several extraction steps are necessary. Urine is strongly absorbed by a polyacrylate powder contained in the absorbent pad. Therefore, addition of salt solutions such as calcium chloride released the absorbed urine by changing the polar environment of the pad. Urine extraction from the diaper was based on a method developed by Liu et al. [37] and further modified by Lucarini et al. [44]. Briefly, the padding of the diaper (14 cm × 6 cm) was cut and mixed in aqueous calcium chloride solution (CaCl_2_ (aq.) at 150 g/L, 150 mL for 15 min) before being filtered (Whatman qualitative filter paper, Grade 1). The recovered liquid was adjusted to pH 5.5 by adding ammonium acetate. The volume of urine extracted from each diaper was quantified by subtracting the final filtered volume to the 150 mL of CaCl_2_ solution used. The total filtered volume (i.e., 150 mL of CaCl_2_ plus the volume of urine from each sample) was divided into 50 mL aliquots. β-Glucuronidase (100 µL) was added, and the solution (50 mL) was incubated overnight at room temperature.

Formic acid (0.1 M, 100 mL) was added, and the solution (150 mL) was extracted with a solid phase extraction (SPE) column (hydrophilic–lipophilic balanced (HLB) SPE column: 3 mL, 200 mg, Macherey-Nagel, Germany). The column was conditioned with 2 mL of methanol followed by formic acid (0.1 M, 2 mL). The sample was loaded onto the SPE and washed with formic acid (0.1 M, 2 mL) followed by deionized water (1 mL). The metabolites were eluted with acetonitrile (3 mL) and ethyl acetate (3 mL). The eluent (6 mL) was evaporated at room temperature under N_2_ flow. The derivatization solution (N,O-bis(trimethylsilyl)trifluoroacetamide (BSTFA) + 1% trimethylchlorosilane (TMCS), 100 µL) was added, and the mixture was incubated for 30 min at 80 °C. The sample was analyzed by gas chromatography–mass spectrometry (GC-MS) analysis. The final phthalate concentrations were adjusted for the total amount of urine quantified for each sample through dilution or concentration factors. 

### 2.5. Quality Assurance and Quality Control

The calibration was carried out by spiking clean disposable diapers with artificial urine (30 mL) (prepared according to Lucarini et al. [44]) with the following concentrations: 0.2, 0.5, 1, 2, 5, 10, 50, 100, and 200 μg/L, (more details are provided in the supplementary material, see Supplementary Materials Figures S1–S9) of the target phthalate metabolites. Blanks were prepared to check possible contamination during sample preparations as well as to exclude the presence of the phthalate monoesters analyzed in unused diapers. Calibration (called internal calibration here) and blank samples with artificial urine or water underwent the same preparation as the urine samples. No phthalate monoesters above the limit of detection (LOD) were detected in any blank samples. In addition to the internal calibration, a direct calibration measurement was performed following the same process as the internal calibration but without adding and extracting artificial urine solution from the diapers. The difference in the measured concentration of the metabolites between the internal and direct calibration was used to calculate the diaper recoveries for each phthalate metabolite absorbed in the diaper (Table 2; Supplementary Materials Table S1). The SPE recovery was calculated between the concentration of the metabolites of the direct calibration before and after the SPE procedure. Different diaper brands (*n* = 23) among the most used in Switzerland were tested. The analysis of artificial urine extracted from diapers of different brands did not show the presence of the investigated phthalates at concentrations higher than the LODs.

### 2.6. Chemical Analytical Analysis

The identification and quantification of the phthalate metabolites were performed by a gas chromatography–mass spectrometer, model QP2010 Ultra from Shimadzu Corporation, Japan equipped with an Rtx-5 amine column (length = 30 m, column diameter = 0.25 mm, column thickness = 0.25 µm), AOC-20i autoinjector and AOC-20s autosampler (Shimadzu Corporation, Japan). The operating software was LabSolutions GC-MS Shimadzu. The carrier gas (helium) flow rate at initial temperature was set at 3 mL/min. Injection temperature was set to 270 °C with splitless mode. The temperature ramp was set at 70 °C and increased to 300 °C at 20 °C/min and then maintained at 300 °C for 8 min. The MS ion source temperature was set at 230 °C. A full scan acquisition mode was used to identify phthalate signals followed by selected ion monitoring mode (SIM) to quantify the target compounds. The SIM method was optimized to quantify the metabolites by analyzing the peaks and their retention times compared to the blanks and by selecting the target fragments (Table 3). 

### 2.7. Statistical Analysis

Statistical analyses were carried out to establish whether or not there were differences in the exposure to phthalates based on gender (Chi-squared test) and age (two-sample *t*-test, testing age with phthalate concentrations >LOD in the urine against age with phthalate concentrations <LOD in the urine). These calculations were performed using R software, as well as the calculations of the standard deviations, means, geometric standard deviations, and geometric means. The mean plus standard deviation plot was performed using GraphPad Prism 9, plotting the age of each child on the y axis against the presence or absence of phthalate metabolite(s) on the *x* axis.

## 3. Results

The total (i.e., sum of free and conjugated) concentration of each metabolite was quantified (Table 4), and at least one of the seven phthalate metabolites was detected in 25 out of the 113 urine samples (22% of positivity >LOD). All these metabolites had remarkably lower detection frequencies with respect to studies reported in Table 5: MEP 11.5%, MBP 3.5%, MCHP 7.1%, MEHP 1.8%, MTMHP 5.3%, MnOP 0.9%, and MBzP 5.3% (Table 4). Among the positive urine samples, 33% were positive for more than one metabolite.

MBP showed the highest arithmetic mean concentration of 74.7 μg/L, while the highest absolute concentration was for MTMHP at 181.5 μg/L. MnOP was detected only in one urine sample. The remaining 78% of the samples did not contain detectable concentrations of any of the phthalate metabolites investigated.

In our study, 25% of the boys (19 out of 76) were positive for at least one of the phthalate metabolites, while 16.2% of the girls (6 out of 37) were positive for at least one. We did not find any significant associations between the age of the children and their urinary phthalate metabolite concentrations (two-sample *t*-test *p*-value = 0.8302). The mean age of the children with a detectable urinary phthalate metabolite was 20.20 months, and the mean age of the children with no detectable phthalate metabolites was 20.51 months.

## 4. Discussion

### 4.1. Phthalate Metabolites Concentrations

This study analyzed for the first time the urine concentrations of phthalate metabolites of very young Swiss children between 6 and 36 months of life. The results suggested that the small cohort investigated was principally exposed to MEP and MCHP with respect to the primary metabolites investigated. Among the samples analyzed, the frequency of detection was surprisingly low, with only 22% of samples containing detectable concentrations of phthalate metabolites.

In 2015, McCombie et al. found a low presence of banned and not-banned phthalates in 88 toys analyzed from the Swiss market [48]. The results obtained by McCombie and colleagues may explain the low detection frequencies that we observed in our study. Indeed, if toys in the Swiss market contain low concentrations of phthalates, young Swiss children may be less exposed to these compounds. Furthermore, these results may also be a sign of the effectiveness of legal restrictions and their application by the industry [48].

In the literature, similar studies involving such a young population are limited. Table 5 shows a comparison of urinary phthalate concentrations observed in the most recent and significant studies [29,30,31,32,33,47].

MEP, the metabolite of DEP used in cosmetics, personal care, washing and cleaning products, and fragrances, is the most frequently detected phthalate in our study. However, this detection frequency is about 6- to 9-fold lower than that observed in other studies (Table 5). On the other hand, we detected a median concentration of MEP that is higher with respect to the findings of similar studies performed in infants, toddlers, and young children (Table 5). For instance, the median concentration of MEP (60.3 μg/L) in this study is 22-fold higher than that observed by Kim et al. (2.7 μg/L) [31].

MCHP was detected in 7.1% of the samples. This compound is a metabolite of DCHP, known to be an endocrine-disrupting chemical (EDC) and specially to affect the reproductive system and the development of reproductive organs [49]. The exposure pattern of infants, toddlers, and young children to DCHP is intriguing. Indeed, DCHP is reported to be mostly used in adhesives, sealants, coating products, paints, inks, toners, or polishes. Comparing to other biomonitoring studies, MCHP was only observed by Liu et al. [29] and only detected in 4.99% of the samples. The mean MCHP concentration was approximately 2000-fold lower than what we found (Table 5).

The MBzP concentration ranges reported by Völkel et al. [33] were comparable to ours, but our detection frequency was lower compared to that of Völkel et al., whilst the same median concentrations were found in our study and by Navaranjan et al. [30] (Table 5). MTMHP, the metabolite of DTMHP, was detected in 5.3% of the samples; however, general knowledge and information about this phthalate are very poor. DTMHP is characterized by C9 (nine carbon) branched chain alkyl di-ester of phthalic acid and can be expected to be included in the group of the isononyl phthalate isomers (DINP). DINP isomers are generally used in vinyl flooring, wire and cable insulation, coated fabrics, gloves, toys, garden hoses, artificial leather, footwear, or in plastic food contact materials [50]. DTMHP, specifically, seems to be an emerging phthalate compound principally used in the manufacturing of tires and liners [51]. Therefore, having detected this phthalate metabolite in 6 out of the 113 subjects of this study is still significant. Similarly, MnOP, the metabolite of DNOP, was detected in one sample at a low concentration (i.e., 2 μg/L). It is not a commonly observed phthalate metabolite, especially among infants, toddlers, and young children, since DNOP is mostly used in tarps, pool liners, flooring tiles, and garden hoses [52].

### 4.2. Population’s Exposure to Phthalates

Recent studies on phthalate toxicokinetics reported elimination half-lives of monoesters such as MEHP and MBP to be a maximum of 6.6 h in adults after ingestion [24,25]. Then, we can estimate that our studied population with low measured concentration(s) of one or more phthalate metabolites were either slightly exposed during the last 24 h or were highly exposed to phthalates earlier. Finally, infants, toddlers, and young children with high concentration(s) of phthalate metabolite(s) may have been exposed during the last 24 h or are likely to be chronically exposed to large amounts of phthalates.

One toddler in our study had higher detectable concentrations with respect to others for four phthalate metabolites: MEP (70.98 μg/L), MBP (75.00 μg/L), MCHP (33.39 μg/L), and MTMHP (16.38 μg/L). We can reasonably assume that this toddler was exposed to DEP, DBP, DCHP, and DTMHP. DEP and DBP are commonly present in personal care products, cosmetics, and washing and cleaning products, as well as PVC items, whereas DCHP and DTMHP are used in the manufacture of paints, sealants, adhesives, or tires.

Two distinct sources of exposure can be envisioned: one is fairly common and predictable for a toddler (DEP, DBP), while the other (DCHP, DTMHP) is more surprising because adults are more likely to be exposed (tire manufacturer, plastics workers, painters) to these two substances. For instance, infants and toddlers may be exposed to phthalates that are mainly used in adult products through breastfeeding as reported by Main et al. [53]. This evidence suggests that infants, toddlers, and young children are exposed through various exposure routes (dermal, oral, inhalative) [54] to phthalates not only directly related to child-specific items but also to items that surround them in everyday life.

### 4.3. Gender-Specific Phthalate Exposure

The proportion of boys who had detectable concentrations of at least one of the tested phthalate metabolites was superior to the proportion of female children with the same result (25% against 16.2%). For MEP, boys had a higher arithmetic mean concentration compared to that of girls (74.46 against 25.63 µg/L), while this was the opposite for MCHP and MBzP (girls had 43.45 and 26.39 µg/L compared to 25.65 and 6.04 µg/L for boys, respectively). We could not compare the other metabolites, as they were not detected in either group. Our results must remain observations and undoubtedly need further investigations due to the small sample size (*n* = 113) and unequal distribution of boys and girls in our cohort (76 boys vs. 37 girls). Sex has been shown to influence the level and type of activities children as early as 3 years old engage with, and several studies have investigated the influence of gender on object-to-mouth and hand-to-mouth behaviors in young children. In an observational study of outdoor activities of 38 children aged 1–6 years old, girls had more frequent and longer hand contacts with certain objects and surfaces compared to boys, but results were not stratified by age [55]. In another observational study of indoor activity patterns of infants and toddlers (6–27 months old) in the farmworker community, boys had greater hand contact frequency with the floor and toys, while girls had longer median mouthing durations with toys and all objects/surfaces [56]. However, two meta-analyses of children approximately 3 months–11 years old revealed that there was not a statistically significant difference in object-to-mouth frequency [57] or hand-to-mouth frequency [58] between genders.

### 4.4. Age-Specific Phthalate Exposure

**Figure 1:**
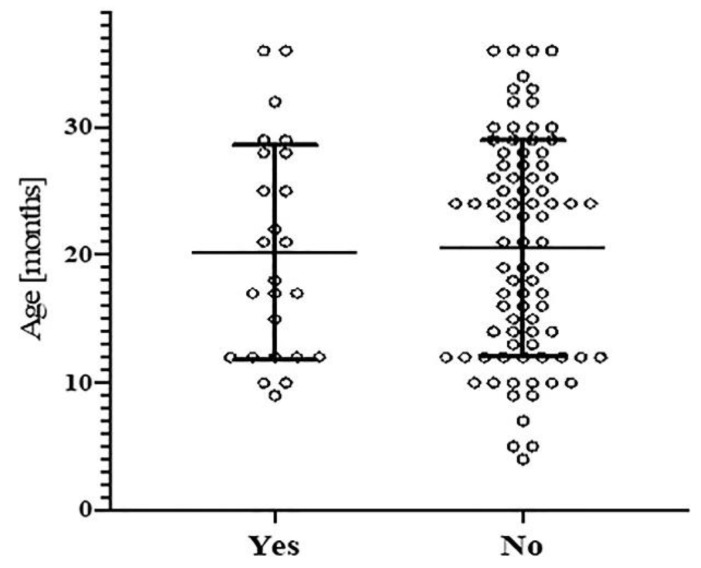
Presence (Yes) or absence (No) of phthalates (concentration > LOD) as a function of the age of the population in months. Mean plus SD plot (months).

There were no significant associations between age and urinary concentrations of phthalate metabolites in our cohort. Moreover, the mean ages of the children with detectable urinary concentrations of phthalate metabolites versus those without were very close (20.2 and 20.51 months of life, Figure 1). It is known that children, and especially young children, have higher (from three to five times) urinary phthalate metabolite levels compared to those in adults [1]. However, scarce data exist for urinary phthalate metabolite levels with respect to developmental changes in children through infancy, childhood, and adolescence. It can be reasonably assumed that infants and toddlers (typically below 2 years old) will have higher urinary phthalate metabolite levels, due to their general mouthing behaviors, compared to older children for whom mouthing activities begin to moderate. Infants (0–1 years old) are typically more exposed to phthalate-containing plasticized toys and childcare articles because of the higher frequency of object-to-mouth exposure (in particular teething), while toddlers (1–2 years old) would probably have more hand-to-mouth exposure [56,59,60]. Phthalates may also be present in dust particles, especially in the indoor environment, so infants, toddlers, and young children may also be exposed through the inhalation route. Infants have a breathing zone that is closer to the floor, while toddlers start to generate their own dust clouds because their mobility on the floor increases. In a study published by Weiss et al. [61], it was shown that exposure by inhalation for a toddler (21 months) was about twice as high as that for adults. However, the inhalation route in toddlers was found to be a minor route of exposure to dust particles containing phthalates compared to ingestion [61].

### 4.5. Regulatory Status of Phthalates in Toys and Childcare Articles

In the European Union, legal restrictions have been implemented under Annex XVII of the Registration, Evaluation, Authorization, and Restriction of Chemicals (REACH) Regulation (EC) No. 1907/2006 [13] for the use of seven phthalates (BBP, DBP, DIBP, DEHP, DIDP, DINP, and DNOP) in toys and childcare articles. In particular, the use of DEHP, BBP, DBP, and DIBP (as substances or in mixtures) is restricted in all children’s toys and childcare articles at concentrations equal to or greater than 0.1% (i.e., 1000 mg/kg) by weight of the plasticized material (REACH, Annex XVII, Entry 51) [16]. This restriction of use also applies to DINP, DIDP, and DNOP but only in toys and childcare articles that can be placed in the mouth by children (REACH, Annex XVII, Entry 52) [15,16,17,18,21]. Similarly, in Switzerland, it is prohibited to place articles on the market if they contain, individually or in any combination, more than 0.1% of DEHP, DIBP, DBP, and BBP in the plasticized material of the article [14]. Similar restrictions have been adopted also by the United States [20] and Canada [19]. These use restrictions were based on evidence of male reproductive toxicity for DEHP, BBP, DBP, and DIBP (classified as Repro. Cat 1B according to CLP Regulation), and on suspected hepatotoxicity in young children of DNOP (classified as Repro. Cat. 2), DINP, and DIDP (which are both not classified as reprotoxicants). Other phthalates such as DEP, DMP, and DCHP are, therefore, used for the production of plastic products.

Comparing our results with the studies reported in Table 5 on similar populations, it appears that there is no decrease in median and GM concentrations of phthalates reported as a result of the implementation of the restrictions. Nevertheless, it appears that our detection frequencies are significantly lower than those found by other studies in Table 5. However, due to the absence in the literature of similar studies conducted on samples collected from 2015 to 2019, it is not possible to establish a real trend on the decrease in detection frequency since the implementation of the regulations.

### 4.6. Limitations and Novelty of the Study

The sample size of this study (*n* = 113) is not large enough to extrapolate the results to the general population of children from 6 months to 3 years old living in Switzerland. Moreover, it should be noted that only spot urine was analyzed for each child, the collection times were not standardized, and the samples were collected during a short and specific time of the year (i.e., summer). Additionally, the medical history and any medications taken by individual children were not known. In future studies, overnight diapers should be collected as they contain more urine than the diapers throughout the day depending on changing schedules. If possible, all diapers should be collected over one day as this would correspond to a 24 h urine sample. Our quantified values are not necessarily linked to a chronic exposure but can also be due to a punctual exposure that happened during the prior 24 h to sampling. However, this study provided for the first time a snapshot of the exposure to phthalates in very young children living in Switzerland. Moreover, this study successfully optimized an analytical method for the quantification of phthalates in urine based on a simple and effective collection medium (i.e., disposable diapers) that can be used in larger investigations.

## 5. Conclusions

Carrying out exposure studies on children who are not toilet trained is challenging because of the difficulty of urine sampling. To overcome this issue, an optimized protocol for urine collection from disposable diapers for the analysis of phthalate metabolites was developed and tested in this study. It was possible with this approach, to quantify internal exposures to seven phthalate metabolites in infants, toddlers, and young children. The study showed limited exposures to phthalates with only 22% of the samples containing these exposure biomarkers. In addition to the most common phthalate metabolites reported previously, we also detected MnOP and MTMHP. These are rarely observed in the urine from infants and toddlers and can, thus, be considered as potentially new emerging phthalates. Considering the vulnerability of infants, toddlers, and young children, comprehensive studies to fully explore the effect of chronic exposures to low concentrations of these phthalates are highly warranted. In addition, further studies on a larger population and over longer periods should be performed to represent the global exposure to phthalates of infants, toddlers, and young children and to understand possible related health effects. In this respect, interlaboratory studies and method validation will be carried out by our research group within the framework of a European program aimed at supporting chemical risk assessment and risk management and promoting partnerships between human biomonitoring laboratories.

## Figures and Tables

**Table 1:** The following phthalates were analyzed in this study: parent phthalates, corresponding monoester metabolites, and CAS numbers.

Phthalate		CAS	Metabolite		CAS
Diethyl phthalate	DEP	84-66-2	Monoethyl phthalate	MEP	2306-33-4
Benzyl butyl phthalate	BBP	85-68-7	Mono-benzyl phthalate	MBzP	2528-16-7
Dibutyl phthalate	DBP	84-74-2	Mono-butyl phthalate	MBP	131-70-4
Dicyclohexyl phthalate	DCHP	84-61-7	Mono-cyclohexyl phthalate	MCHP	7517-36-4
Di-2-ethylhexyl phthalate	DEHP	117-81-7	Mono-2-ethylhexyl phthalate	MEHP	4376-20-9
Bis(3,5,5-trimethylhexyl) phthalate	DTMHP	14103-61-8	Mono(3,5,5-trimethylhexyl) phthalate	MTMHP	297182-83-3
Di-n-octyl phthalate	DNOP	117-84-0	Mono-n-octyl phthalate	MnOP	5393-19-1

**Table 2:** Mean diaper and SPE recovery for each derivatized phthalate metabolite at concentrations 10, 50, and 100 μg/L; limit of detection (LOD) and limit of quantification (LOQ) in μg/L.

Derivatized Phthalate Metabolite	Diaper Recovery (%)	SPE Recovery (%)	LOD	LOQ
MEP	135%	98%	0.20	0.61
MBP	116%	77%	0.17	0.52
MCHP	124%	96%	0.17	0.52
MEHP	89%	99%	0.19	0.54
MTMHP	66%	94%	0.12	0.38
MnOP	68%	89%	0.26	0.80
MBzP	117%	115%	0.18	0.56

**Table 3:** Fragments (quantifier and qualifier ions) and retention times of the phthalate metabolites used as parameters for the GC-MS SIM method.

Derivatized Phthalate Metabolite	Quantifier Ion, *Qualifier Ion(s) [m/z]*	Retention Time [min]
MEP	75.00; *73.00, 223.00*	8.325
MBP	75.00; *73.00, 223.00*	9.400
MCHP	221.00; *75.00, 221.00*	10.850
MEHP	221.00; *75.00, 221.00*	10.950
MTMHP	57.00; *73.00, 221.00*	11.200
MnOP	221.00; *73.00, 223.00*	11.400
MBzP	91.00; *73.00, 179.00*	11.475

**Table 4:** Concentration range for urine samples with quantifiable metabolites (>LOQ) in μg/L; arithmetic mean (AM and standard deviation (SD)); geometric mean (GM and geometric standard deviation (GSD)); number of samples with concentration of metabolites >LOD; and detection frequency (DF) in %.

Metabolites	Conc. Range	AM (SD)	GM (GSD)	*n* of Samples	DF%
MEP	3.4–142.9	63.2 (51.6)	36.1 (3.7)	13	11.5
MBP	10.8–117.4	74.7 (46.0)	54.9 (3.0)	4	3.5
MCHP	2.3–55.9	27.9 (20.3)	18.2 (3.2)	8	7.1
MEHP	30.9	-	-	2	1.8
MTMHP	16.4–181.5	55.0 (70.9)	34.5 (2.6)	6	5.3
MnOP	2.0	-	-	1	0.9
MBzP	4.4–47.8	12.8 (17.2)	7.9 (2.5)	6	5.3

**Table 5:** Recent biomonitoring studies on the exposure of infants, toddlers, and young children to the seven phthalate metabolites investigated in this study (median, mean, and GM concentration expressed in μg/L). For the sake of clarity, only the metabolites analyzed in this study have been reported in the table.

Country (Collection Year)	*n*	Months of Age		MEHP	MEP	MBP	MBzP	MCHP	MnOP	MTMHP	Ref.
Finland (2006–2009)	432	1–14	Median Range DF (%)	0.44 <LOD–849 76.2%	10.7 0.82–486 100%	15.2 <LOD–156 99.8%	24.6 1.85–1985 100%				[32]
Germany (2008)	207	1–5	Median Range DF (%)	n.d. <1–19.1 15%	12.1 <2.5–224.5 78%	11.5 2.6–88.5 99.5%	2.5 2.5–40.4 50%				[33]
Korea (2012–2013)	268	3–15	Median (IQR) * DF (%)		2.71 97%	12.41 99%					[31]
Sweden (2009–2012)	83	2–6	Median Range DF (%)		19.6 2.7–270.4 100%	39.1 4.7–315.5 100%	10.5 0.4–194.9 100%				[47]
Canada (2008–2012)	1072	12	Median GM (GSD) Max Value DF (%)	1.7 1.8 (2.4) 96 83.8%	11.4 12.4 (3.3) 5210 95.8%	22.9 24.3 (2.2) 1002 99.7%	4.9 5.6 (3.7) 329 87.9%				[30]
Canada (2008–2012)	898	36	Median GM (GSD) Max Value DF (%)	2.2 2.2 (2.3) 143 77.7%	12.6 13.9 (3.0) 1804 91.1%	31.7 32.1 (2.2) 1144 98.6%	7.6 8.5 (3.4) 1071 87.3%				[30]
China (2012–2014)	155	0–12	Mean GM Range DF (%)	5.19 2.51 <LOD–193 98.8%	16.8 4.01 <LOD–2399 99.9%	22.2 10.1 <LOD–780.7 99.8%	0.26 0.12 <LOD–27.2 60.0%	0.007 0.05 <LOD–0.59 4.99%			[29]
Switzerland (2019)	113	6–36	GM Median Range DF (%)	n.d. - <LOD–30.9 1.8%	36.1 60.3 3.4–142.9 11.5%	54.9 85.3 10.8–117.4 3.5%	7.9 4.9 4.4–47.8 5.3%	18.2 27.3 2.3–55.9 7.1%	n.d. - 2 0.9%	34.5 27.8 <LOD–181.5 5.3%	This study

Abbreviations: IQR, interquartile range; DF, detection frequency %; n.d., not determined.

## Data Availability

Data available on request.

## Supplementary Materials

The following are available online at https://www.mdpi.com/article/10.3390/ijerph18136806/s1, Figure S1: (a) calibration curve for MEP, TMS derivative; (b) GC-MS chromatograms for the quantifier ion at the calibration points, Figure S2: (a) calibration curve for MBP, TMS derivative; (b) GC-MS chromatograms for the quantifier ion at the calibration points, Figure S3: (a) calibration curve for MCHP, TMS derivative; (b) GC-MS chromatograms for the quantifier ion at the calibration points, Figure S4: (a) calibration curve for MEHP, TMS derivative; (b) GC-MS chromatograms for the quantifier ion at the calibration points, Figure S5: (a) calibration curve for MTMHP, TMS derivative; (b) GC-MS chromatograms for the quantifier ion at the calibration points, Figure S6: (a) calibration curve for MnOP, TMS derivative; b) GC-MS chromatograms for the quantifier ion at the calibration points, Figure S7: (a) calibration curve for MBzP, TMS derivative; (b) GC-MS chromatograms for the quantifier ion at the calibration points, Figure S8: GC-MS chromatogram of the calibration curve passed through the diaper at 50 mg/L. Only quantifier ions are shown for clarity, Figure S9: Example of Chromatograms with detectable concentrations of (a) MBzP, (b) MCHP, (c) MTMHP, Table S1: ranges of recovery for each metabolite.

## References

[1] Becker K., Göen T., Seiwert M., Conrad A., Pick-Fuß H., Müller J., Wittassek M., Schulz C., Kolossa-Gehring M. (2009). GerES IV: Phthalate metabolites and bisphenol A in urine of German children. Int. J. Hyg. Environ. Health.

[2] Chanda M., Roy S.K. (2008). Industrial Polymers, Specialty Polymers, and Their Applications. Plastics Fundamentals, Properties, and Testing.

[3] Radke E., Galizia A., Thayer K.A., Cooper G.S. (2019). Phthalate exposure and metabolic effects: A systematic review of the human epidemiological evidence. Environ. Int..

[4] Benjamin S., Masai E., Kamimura N., Takahashi K., Anderson R.C., Faisal P.A. (2017). Phthalates impact human health: Epidemiological evidences and plausible mechanism of action. J. Hazard. Mater..

[5] Franken C., Lambrechts N., Govarts E., Koppen G., Hond E.D., Ooms D., Voorspoels S., Bruckers L., Loots I., Nelen V. (2017). Phthalate-induced oxidative stress and association with asthma-related airway inflammation in adolescents. Int. J. Hyg. Environ. Health.

[6] Qian X., Li J., Xu S., Wan Y., Li Y., Jiang Y., Zhao H., Zhou Y., Liao J., Liu H. (2019). Prenatal exposure to phthalates and neurocognitive development in children at two years of age. Environ. Int..

[7] Braun J.M., Sathyanarayana S., Hauser R. (2013). Phthalate exposure and children’s health. Curr. Opin. Pediatr..

[8] Radke E.G., Braun J.M., Meeker J.D., Cooper G.S. (2018). Phthalate exposure and male reproductive outcomes: A systematic review of the human epidemiological evidence. Environ. Int..

[9] Radke E.G., Glenn B.S., Braun J.M., Cooper G.S. (2019). Phthalate exposure and female reproductive and developmental outcomes: A systematic review of the human epidemiological evidence. Environ. Int..

[10] Radke E., Braun J.M., Nachman R.M., Cooper G.S. (2020). Phthalate exposure and neurodevelopment: A systematic review and meta-analysis of human epidemiological evidence. Environ. Int..

[11] Hauser R., Calafat A.M. (2005). Phthalates and human health. Occup. Environ. Med..

[12] Wang Y., Zhu H., Kannan K. (2019). A Review of Biomonitoring of Phthalate Exposures. Toxics.

[13] EC (European Commission) (2006). Regulation (EC) No 1907/2006 of the European Parliament and of the Council of 18 December 2006 concerning the Registration, Evaluation, Authorisation and Restriction of Chemicals (REACH), establishing a European Chemicals Agency, amending Directive 1999/45/EC and repealing Council Regulation(EEC) No 793/93 and Commission Regulation (EC) No 1488/94 as well as Council Directive 76/769/EEC and Commission Directives 91/155/EEC, 93/67/EEC, 93/105/EC and 2000/21/EC. Off. J. Eur. Union L.

[14] Ordinance on the Reduction of Risks Relating to the Use of Certain Particularly Dangerous Substances, Preparations and Articles (Chemical Risk Reduction Ordinance, ORRChem) of 18 May 2005 (Status as of 1 March 2021). https://www.fedlex.admin.ch/eli/cc/2005/478/en#app26.

[15] ECHA (European Chemicals Agency) Annex XVII to Reach, Entry 52. Conditions of Restrictions on the Manufacture, Placing on the Market and Use of Certain Dangerous Substances, Mixtures and Articles. https://echa.europa.eu/documents/10162/57096439-2ddd-4f14-b832-85181a09f595.

[16] ECHA (European Chemicals Agency) Annex XVII to Reach, Entry 51. Conditions of Restrictions on the Manufacture, Placing on the Market and Use of Certain Dangerous Substances, Mixtures and Articles. https://echa.europa.eu/documents/10162/aaa92146-a005-1dc2-debe-93c80b57c5ee.

[17] EC (European Commission) (2009). Commission Regulation (EC) No 552/2009 Of 22 June 2009 Amending Regulation (EC) No 1907/2006 of the European Parliament and of the Council on the Registration,Evaluation, Authorisation and Restriction of Chemicals (REACH) as Regards Annex XVII (Text with EEA Relevance). Off. J. Eur. Union..

[18] EC (European Commission) (2015). Directives Commission Delegated Directive (EU) 2015/863 of 31 March 2015 Amending Annex II to DIRECTIVE 2011/65/EU of the European Parliament and of the Council as Regards the List of Restricted Substances (Text with EEA Relevance). Off. J. Eur. Union..

[19] Health Canada (2016). Industry Guide to Health Canada’s Safety Requirements for Children’s Toys and Related Products.

[20] CPSC, Consumer Product Safety Commission (2017). Prohibition of Children’s Toys and Child Care Articles Containing Specified Phthalates: Determinations Regarding Certain Plastics. Federal Register, Docket No. CPSC-2016-0017. https://www.federalregister.gov/documents/2016/08/17/2016-19464/prohibition-of-childrens-toys-and-child-care-articles-containing-specified-phthalates-determinations.

[21] EC (European Commission) (2018). Commission Regulation (EU) 2018/2005 of 17 December 2018 Amending Annex XVII to REGULATION (EC) No 1907/2006 of the European Parliament and of the Council Concerning the Registration, Evaluation, Authorisation and Restriction of Chemicals (REACH) as Regards Bis(2-ethylhexyl) Phthalate (DEHP), Dibutyl Phthalate (DBP), Benzyl Butyl Phthalate (BBP) and Diisobutyl Phthalate (DIBP) (Text with EEA Relevance). Off. J. Eur. Union.

[22] Oteef M.D.Y., Elhassan M.S. (2020). Plastic toys and child care articles as a source of children exposure to phthalates and other plasticisers in Saudi Arabia. Int. J. Environ. Anal. Chem..

[23] Schwedler G., Rucic E., Lange R., Conrad A., Koch H.M., Pälmke C., Brüning T., Schulz C., Schmied-Tobies M.I., Daniels A. (2020). Phthalate metabolites in urine of children and adolescents in Germany. Human biomonitoring results of the German Environmental Survey GerES V, 2014–2017. Int. J. Hyg. Environ. Health.

[24] Kessler W., Numtip W., Völkel W., Seckin E., Csanády G.A., Pütz C., Klein D., Fromme H., Filser J.G. (2012). Kinetics of di(2-ethylhexyl) phthalate (DEHP) and mono(2-ethylhexyl) phthalate in blood and of DEHP metabolites in urine of male volunteers after single ingestion of ring-deuterated DEHP. Toxicol. Appl. Pharmacol..

[25] Mittermeier A., Völkel W., Fromme H. (2016). Kinetics of the phthalate metabolites mono-2-ethylhexyl phthalate (MEHP) and mono-n-butyl phthalate (MnBP) in male subjects after a single oral dose. Toxicol. Lett..

[26] Kumar A.R., Sivaperumal P. (2016). Analytical methods for the determination of biomarkers of exposure to phthalates in human urine samples. TrAC Trends Anal. Chem..

[27] Frederiksen H., Skakkebaek N.E., Andersson A.-M. (2007). Metabolism of phthalates in humans. Mol. Nutr. Food Res..

[28] Ginsberg G., Hattis D., Sonawane B. (2004). Incorporating pharmacokinetic differences between children and adults in assessing children’s risks to environmental toxicants. Toxicol. Appl. Pharmacol..

[29] Liu L., Wang H., Li X., Tian M., Huang Q., Zhang J., Pan H., Wen K., Huang Q., Yan J. (2020). Infantile phthalate metabolism and toxico/pharmacokinetic implications within the first year of life. Environ. Int..

[30] Navaranjan G., Takaro T.K., Wheeler A.J., Diamond M.L., Shu H., Azad M.B., Becker A.B., Dai R., Harris S.A., Lefebvre D.L. (2019). Early life exposure to phthalates in the Canadian Healthy Infant Longitudinal Development (CHILD) study: A multi-city birth cohort. J. Expo. Sci. Environ. Epidemiol..

[31] Kim S., Lee J., Park J., Kim H.-J., Cho G.J., Kim G.-H., Eun S.-H., Lee J.J., Choi G., Suh E. (2017). Urinary phthalate metabolites over the first 15 months of life and risk assessment – CHECK cohort study. Sci. Total Environ..

[32] Frederiksen H., Kuiri-Hänninen T., Main K.M., Dunkel L., Sankilampi U. (2014). A Longitudinal Study of Urinary Phthalate Excretion in 58 Full-Term and 67 Preterm Infants from Birth through 14 Months. Environ. Health Perspect..

[33] Völkel W., Kiranoglu M., Schuster R., Fromme H. (2014). Phthalate intake by infants calculated from biomonitoring data. Toxicol. Lett..

[34] Song N.R., On J.-W., Lee J., Park J.-D., Kwon H.-J., Yoon H.J., Pyo H. (2013). Biomonitoring of urinary di(2-ethylhexyl) phthalate metabolites of mother and child pairs in South Korea. Environ. Int..

[35] Hond E.D., Govarts E., Willems H., Smolders R., Casteleyn L., Kolossa-Gehring M., Schwedler G., Seiwert M., Fiddicke U., Castaño A. (2015). First Steps toward Harmonized Human Biomonitoring in Europe: Demonstration Project to Perform Human Biomonitoring on a European Scale. Environ. Health Perspect..

[36] CDC (2012). Centers for Diseas Control and Prevention, 2012. Fourth Annual Report. https://www.cdc.gov/exposurereport/pdf/fourthreport.pdf.

[37] Liu L., Xia T., Guo L., Cao L., Zhao B., Zhang J., Dong S., Shen H. (2012). Expressing urine from a gel disposable diaper for biomonitoring using phthalates as an example. J. Expo. Sci. Environ. Epidemiol..

[38] Hu Y., Beach J., Raymer J., Gardner M. (2004). Disposable diaper to collect urine samples from young children for pyrethroid pesticide studies. J. Expo. Sci. Environ. Epidemiol..

[39] Oya N., Ito Y., Hioki K., Asai Y., Aoi A., Sugiura Y., Ueyama J., Oguri T., Kato S., Ebara T. (2017). Quantitative analysis of organophosphate insecticide metabolites in urine extracted from disposable diapers of toddlers in Japan. Int. J. Hyg. Environ. Health.

[40] Ueyama J., Aoi A., Ueda Y., Oya N., Sugiura Y., Ito Y., Ebara T., Kamijima M. (2020). Biomonitoring method for neonicotinoid insecticides in urine of non-toilet-trained children using LC-MS/MS. Food Addit. Contam. Part A.

[41] Saito S., Ueyama J., Kondo T., Saito I., Shibata E., Gotoh M., Nomura H., Wakusawa S., Nakai K., Kamijima M. (2013). A non-invasive biomonitoring method for assessing levels of urinary pyrethroid metabolites in diapered children by gas chromatography–mass spectrometry. J. Expo. Sci. Environ. Epidemiol..

[42] Oerlemans A., van Dael M., Vermeulen R., Russel F., Scheepers P. (2018). Urine collection methods for non-toilet-trained children in biological monitoring studies: Validation of a disposable diaper for characterization of tebuconazole exposure. Toxicol. Lett..

[43] Liu L., Xia T., Zhang X., Barr D.B., Alamdar A., Zhang J., Tian M., Huang Q., Shen H. (2014). Biomonitoring of infant exposure to phenolic endocrine disruptors using urine expressed from disposable gel diapers. Anal. Bioanal. Chem..

[44] Lucarini F., Krasniqi T., Rosset G.B., Roth N., Hopf N.B., Broillet M.-C., Staedler D. (2020). Exposure to New Emerging Bisphenols Among Young Children in Switzerland. Int. J. Environ. Res. Public Health.

[45] Silva M.J., Samandar E., Preau J.L., Needham L.L., Calafat A.M. (2006). Urinary oxidative metabolites of di(2-ethylhexyl) phthalate in humans. Toxicology.

[46] Hubal E.A.C., de Wet T., Du Toit L., Firestone M.P., Ruchirawat M., van Engelen J., Vickers C. (2014). Identifying important life stages for monitoring and assessing risks from exposures to environmental contaminants: Results of a World Health Organization review. Regul. Toxicol. Pharmacol..

[47] Carlstedt F., Jönsson B., Bornehag C.-G. (2012). PVC flooring is related to human uptake of phthalates in infants. Indoor Air.

[48] McCombie G., Biedermann S., Suter G., Biedermann M. (2017). Survey on plasticizers currently found in PVC toys on the Swiss market: Banned phthalates are only a minor concern. J. Environ. Sci. Health Part A.

[49] Ahbab M.A., Barlas N. (2015). Influence of in utero di-n-hexyl phthalate and dicyclohexyl phthalate on fetal testicular development in rats. Toxicol. Lett..

[50] California Environmental Protection Agency, Office of Environmental Health Hazard Assessment (2013). Evidence of the carcinogenicity of diisononyl phthalate (DINP). Oehha.ca.gov/media/downloads/proposition-65/chemicals/dinphid100413.pdf.

[51] Shun S., Shusaku T., Hirota K. Thermoplastic Resin Composition for Tire Inner Liner, Tire Inner Liner, Pneumatic Tire, Manufacturing Method of Tire Inner Liner, and Manufacturing Method of Pneumatic Tire. JP2019182969 (A), Japan Office. Publication Date: 24-10-2019. https://patentscope.wipo.int/search/en/detail.jsf?docId=JP275662608&tab=NATIONALBIBLIO&_cid=P21-KO2M88-10574-1.

[52] CPSC, Consumer Product Safety Commission (2010). Toxicity Review of Di-n-Octyl Phthalate (DnOP).

[53] Main K.M., Mortensen G.K., Kaleva M.M., Boisen K.A., Damgaard I.N., Chellakooty M., Schmidt I.M., Suomi A.-M., Virtanen H.E., Petersen D.V.H. (2006). Human Breast Milk Contamination with Phthalates and Alterations of Endogenous Reproductive Hormones in Infants Three Months of Age. Environ. Health Perspect..

[54] Wormuth M., Scheringer M., Vollenweider M., Hungerbühler K. (2006). What are the sources of exposure to eight frequently used phthalic acid esters in Europeans?. Risk Anal..

[55] Auyeung W., Canales R.A., Beamer P., Ferguson A.C., Leckie J.O. (2006). Young children’s hand contact activities: An observational study via videotaping in primarily outdoor residential settings. J. Expo. Sci. Environ. Epidemiol..

[56] Beamer P., Key M.E., Ferguson A., Canales R.A., Auyeung W., Leckie J.O. (2008). Quantified activity pattern data from 6 to 27-month-old farmworker children for use in exposure assessment. Environ. Res..

[57] Xue J., Zartarian V., Tulve N., Moya J., Freeman N., Auyeung W., Beamer P. (2010). A meta-analysis of children’s object-to-mouth frequency data for estimating non-dietary ingestion exposure. J. Expo. Sci. Environ. Epidemiol..

[58] Xue J., Zartarian V., Moya J., Freeman N., Beamer P., Black K., Tulve N., Shalat S. (2007). A meta-analysis of children’s hand-to-mouth frequency data for estimating nondietary ingestion exposure. Risk Anal..

[59] Tulve N.S., Suggs J.C., McCurdy T., Hubal E.A.C., Moya J. (2002). Frequency of mouthing behavior in young children. J. Expo. Sci. Environ. Epidemiol..

[60] Farooq T., Hameed A., Raza A., Akash M.S.H., Rehman K., Hashmi M.Z. (2021). Role of Phthalates as EDCs in Metabolic Disorders, in Endocrine Disrupting Chemicals-Induced Metabolic Disorders and Treatment Strategies.

[61] Weiss J.M., Gustafsson Å., Gerde P., Bergman Å., Lindh C.H., Krais A.M. (2018). Daily intake of phthalates, MEHP, and DINCH by ingestion and inhalation. Chemosphere.

