# Supplementary material for: Concentrations of Seven Phthalate Monoesters in Infants and Toddlers Quantified in Urine Extracted from Diapers
Source: Int J Environ Res Public Health. 2021 Jun 24;18(13):6806. doi: 10.3390/ijerph18136806 (PMC8297146; doi:10.3390/ijerph18136806)
Supplement: Supplementary file 1 [file ijerph-18-06806-s001.zip › Supplementary Material.pdf]

Supplementary Material:

Concentrations of Seven Phthalate Monoesters in Infants and Toddlers Quantified in Urine Extracted from Diapers

Fiorella Lucarini [1], Marc Blanchard [1], Tropoja Krasniqi [1], Nicolas Duda [1], Gaëlle Bailat Rosset [2], Alessandro Ceschi [3,4,5], Nicolas Roth [6], Nancy B. Hopf [6,7], Marie-Christine Broillet [1] and Davide Staedler [1,2,*]

1 Department of Biomedical Sciences, University of Lausanne, 1011 Lausanne, Switzerland; fiorella.lucarini@unil.ch (F.L.); marc.blanchard@unil.ch (M.B.); tropoja.krasniqi@unil.ch (T.K.); nicolas.duda@unil.ch (N.D.); marie-christine.broillet@unil.ch (M.-C.B.)
2 Scitec Research SA, Av. De Provence 18, 1007 Lausanne, Switzerland; gbailat@scitec-research.com
3 Division of Clinical Pharmacology and Toxicology, Institute of Pharmacological Sciences of Southern Switzerland, Ente Ospedaliero Cantonale, 6900 Lugano, Switzerland
4 Faculty of Biomedical Sciences, Università della Svizzera Italiana, 6900 Lugano, Switzerland
5 Department of Clinical Pharmacology and Toxicology, University Hospital Zurich, 8091 Zurich, Switzerland
6 Swiss Centre for Applied Human Toxicology (SCAHT), University of Basel, 4055 Basel, Switzerland; nicolas.roth@unibas.ch (N.R.); nancy.hopf@unisante.ch (N.B.H.)
7 Center for Primary Care and Public Health (Unisanté), University of Lausanne, 1007 Lausanne, Switzerland
* Correspondence: davide.staedler@unil.ch; Tel.: +41-(0)-79-702-1423

Calibration curves and chromatograms

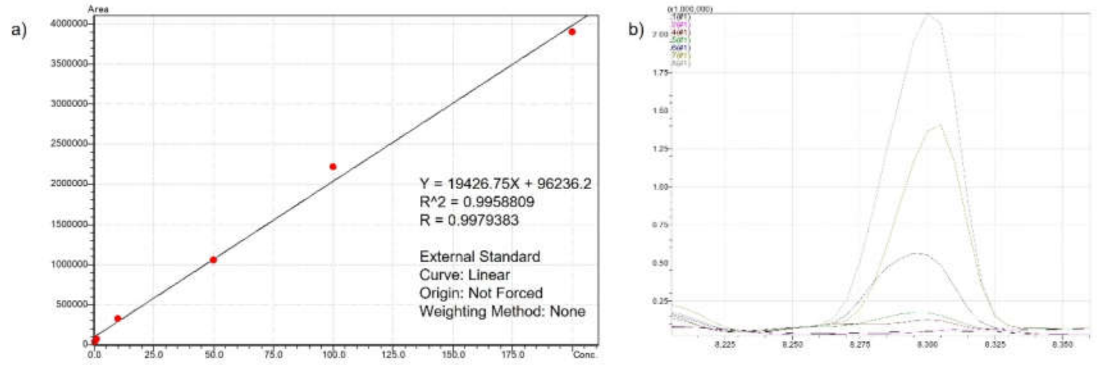

Figure S1: a) calibration curve for MEP, TMS derivative; b) GC-MS chromatograms for the quantifier ion at the calibration points.

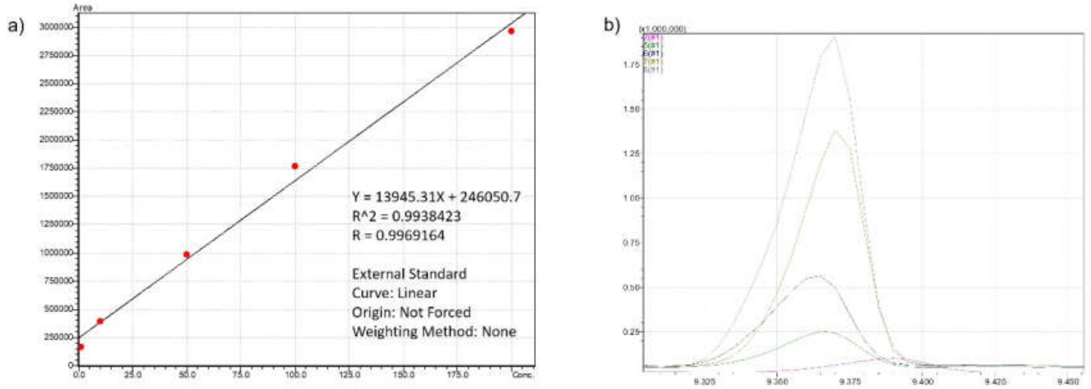

Figure S2: a) calibration curve for MBP, TMS derivative; b) GC-MS chromatograms for the quantifier ion at the calibration points.

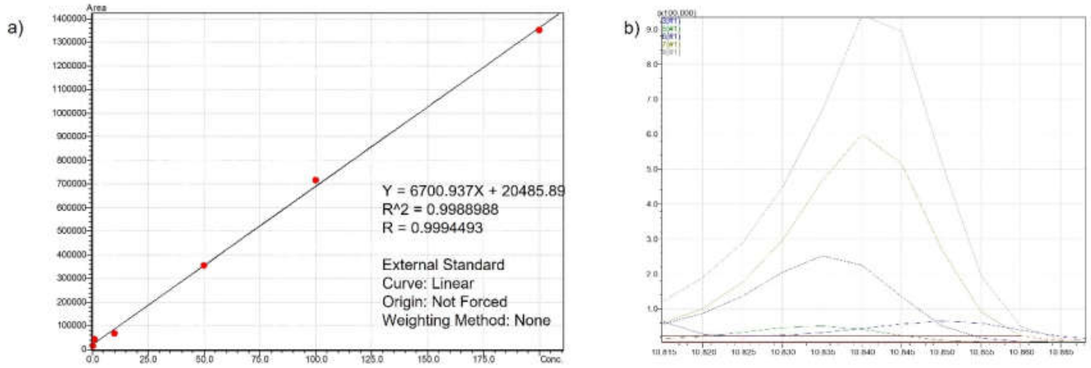

Figure S3: a) calibration curve for MCHP, TMS derivative; b) GC-MS chromatograms for the quantifier ion at the calibration points.

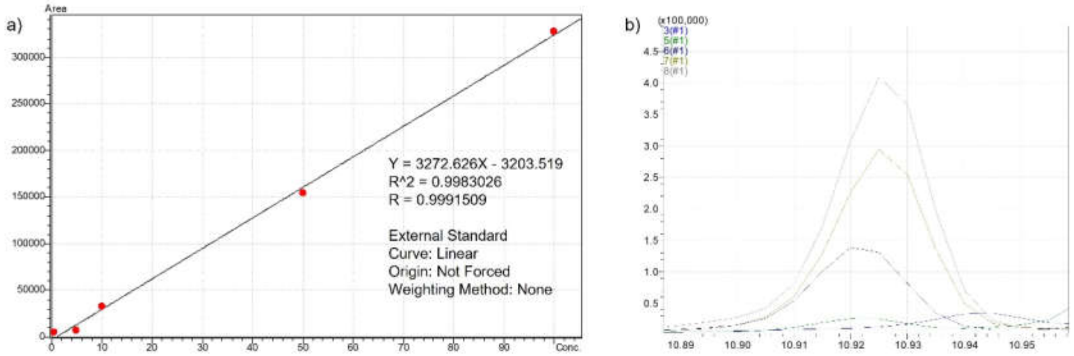

Figure S4: a) calibration curve for MEHP, TMS derivative; b) GC-MS chromatograms for the quantifier ion at the calibration points.

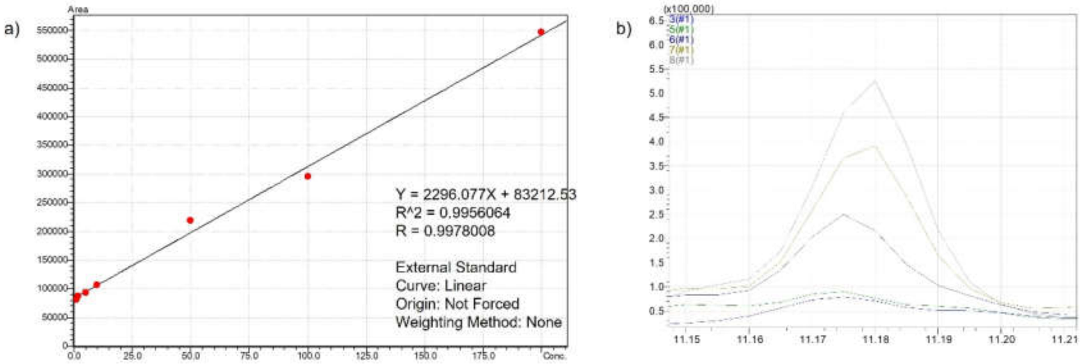

Figure S5: a) calibration curve for MTMHP, TMS derivative; b) GC-MS chromatograms for the quantifier ion at the calibration points.

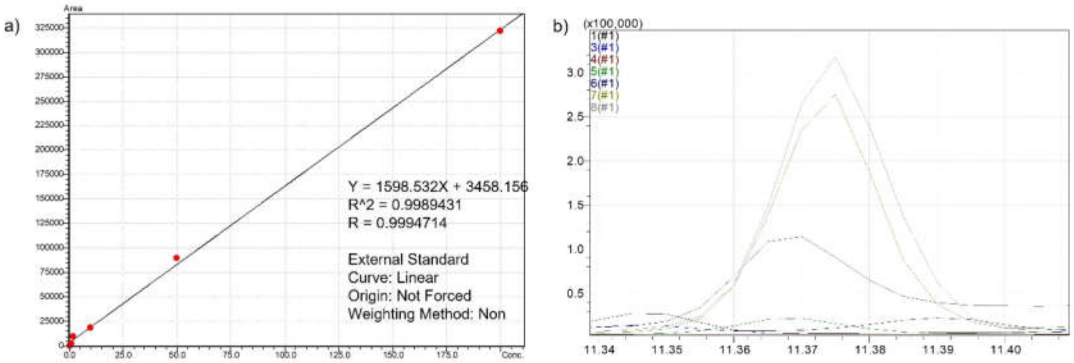

Figure S6: a) calibration curve for MnOP, TMS derivative; b) GC-MS chromatograms for the quantifier ion at the calibration points.

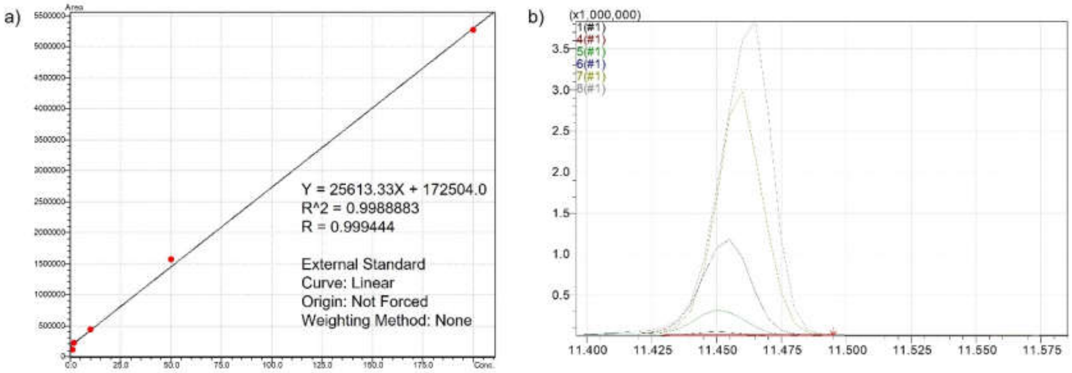

Figure S7: a) calibration curve for MBzP, TMS derivative; b) GC-MS chromatograms for the quantifier ion at the calibration points

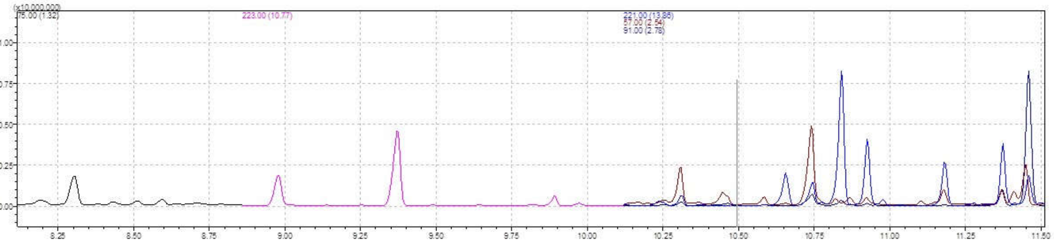

Figure S8: GC-MS chromatogram of the calibration curve passed through the diaper at 50 mg/L. Only quantifier ions are shown for clarity.

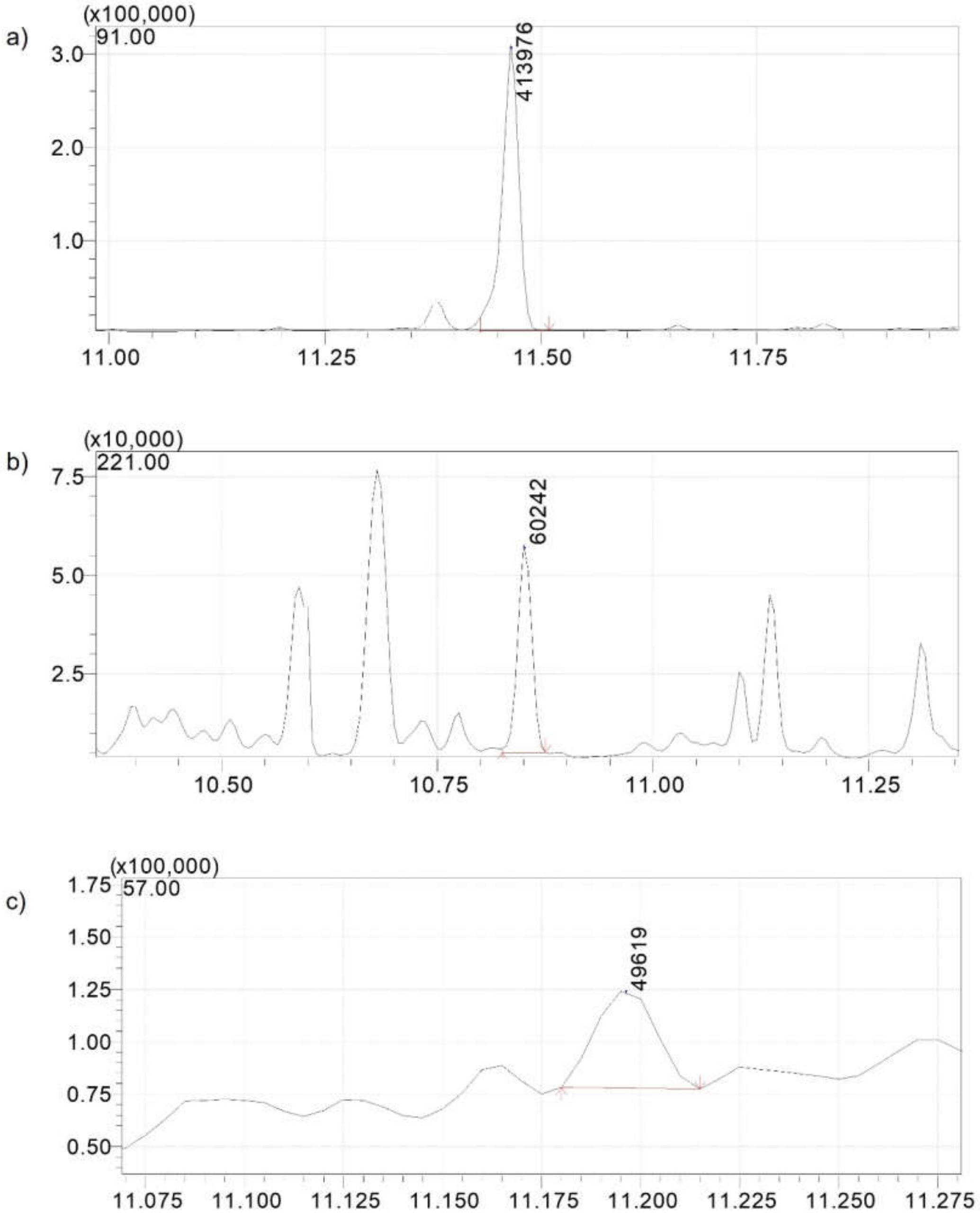

Figure S9: Example of Chromatograms with detectable concentrations of a) MBzP, b) MCHP, c) MTMHP.

Table S1: Ranges of recovery for each metabolite.

	Diaper Recoveries %		
**Derivatized phthalate metabolite**	**10mg/L**	**50mg/L**	**100 mg/L**
**MEP**	130	134	140
**MBP**	120	129	98
**MCHP**	129	116	126
**MEHP**	87	96	85
**MTMHP**	67	67	65
**MnOP**	64	62	79
**MBzP**	118	109	124
